# Clinical and serologic features of sicca syndrome in a tertiary care cohort in China: a retrospective rheumatologic study

**DOI:** 10.3389/fmed.2026.1935003

**Published:** 2026-10-27

**Authors:** Yanying Zhang, Zhigang Sun, Jiayu Zhang, Chang Su, Le Sun, Shujuan Chen, Sihui He, Chunzhi Tang

**Affiliations:** 1 School of Acupuncture, Moxibustion and Rehabilitation, Guangzhou University of Chinese Medicine, Guangzhou, Guangdong, China; 2 Department of Rheumatology, Shenzhen Traditional Chinese Medicine Hospital, Shenzhen, Guangdong, China; 3 Shenzhen Pingle Orthopedics Hospital (Pingshan District Hospital of Traditional Chinese Medicine), Shenzhen, Guangdong, China; 4 Guangzhou Health Science College, Guangzhou, Guangdong, China

**Keywords:** anti-SSA/Ro, autoantibodies, autoimmune disease, extraglandular manifestations, rheumatology, serologic markers, sicca syndrome

## Abstract

**Background:**

Sicca syndrome is a clinical syndrome with heterogeneous pathogenesis defined by chronic ocular and/or oral dryness resulting from autoimmune disorders, medications, ageing or other systemic disorders. The clinical and serologic spectrum must be clearly defined to enable accurate diagnostic assessment and clinical characterization.

**Objective:**

To assess the clinical and serologic characteristics of patients with sicca syndrome and identify factors associated with extraglandular manifestations.

**Methodology:**

A retrospective observational study of 765 adults with sicca syndrome who were treated at a tertiary care rheumatology centre in China from January 2023 to December 2025. Information was retrieved from the electronic medical record, which included demographic, clinical, laboratory, and autoantibody data. Data analysis was performed using SPSS version 29.0, with comparative analysis, correlation tests, multivariable logistic regression and sensitivity analysis of the regression model.

**Results:**

The average age was 52.8 ± 13.6 years and 86.7% of patients were female. 70% of patients had both ocular and oral dryness, and 59.1% had at least one extraglandular manifestation. ANA, anti-SSA/Ro, anti-SSB/La and rheumatoid factor were positive in 74.6%, 61.2%, 36.1% and 44.7% of patients, respectively. Patients who were anti-SSA positive had significantly more systemic involvement, compared to those who were anti-SSA negative (65.2% vs. 49.5%, *P* < 0.001).

**Conclusion:**

The findings of this tertiary-care cohort were dominated by middle-aged women with sicca syndrome, and there was significant clinical and serologic variability. Anti-SSA antibodies, hypergammaglobulinemia, increased inflammatory markers and complement consumption were associated with systemic manifestations. Anti-SSA/Ro positivity may be a marker of a distinct serological and potentially systemic phenotype; however, given the observational design, these findings reflect associations rather than causal relationships and may inform, rather than determine, the clinical assessment of patients with sicca syndrome.

## 1 Introduction

Sicca syndrome is a heterogeneous clinical condition characterised by persistent ocular and/or oral dryness resulting from impaired lacrimal and salivary gland function. These symptoms may occur in association with ageing, medications, systemic conditions, or autoimmune diseases, particularly primary Sjögren's syndrome (pSS), which is a major autoimmune exocrinopathy (1). The chronicity of sicca symptoms significantly impairs oral function, vision, nutrition, and quality of life; therefore, early detection and diagnosis are of great importance in the rheumatology clinic. The clinical features of the sicca syndrome can be highly variable, ranging from involvement of a single gland to involvement of multiple organs (2). Patients may have varying symptoms and clinical features, and a multimodal assessment combining clinical examination, laboratory tests, imaging, and histopathological analysis is essential for accurate characterisation of the disease (3).

Moreover, immunophenotyping has helped distinguish autoimmune Sjögren's from non-autoimmune sicca syndrome, thereby improving diagnostic accuracy (4). Furthermore, detailed profiling of anti-Ro52, anti-Ro60, and anti-La antibodies has helped improve understanding of the serological diversity and disease heterogeneity of anti-Ro antibody disease (5). Sicca symptoms may precede the fulfilment of classification criteria for primary Sjögren's syndrome, and some patients with isolated dryness may meet these criteria only after prolonged follow-up (6). On the other hand, patients with non-Sjögren's sicca may experience substantial physical and oral health problems without having an underlying autoimmune disease (7). These observations highlight the importance of comprehensive clinical assessment regardless of the underlying aetiology. In addition to glandular involvement, systemic involvement can significantly increase disease burden and exacerbate functional outcomes (8).

More recently, symptom-based clustering techniques have been used to categorise patients into clinically relevant phenotypes based on the presence of dryness, fatigue, and pain, thereby helping guide personalised treatment approaches (9). Other environmental and immunological factors also contribute to disease expression. There is an association between smoking and differences in disease classification and clinical phenotype (10), and tear cytokine profiling has become a promising approach to assess sicca-related dry eye (11).

Similarly, the close link between oral and ocular dryness underscores the need for an integrated assessment of exocrine gland dysfunction (12). More recently, a combined clinical and immunological evaluation (13) was again highlighted, as the value of serological characteristics in predicting the severity of dry eye manifestations was demonstrated in the Sjögren's International Collaborative Clinical Alliance (SICCA) cohort. Nevertheless, there are few studies reporting the clinical and serological features of Chinese patients with sicca syndrome in tertiary rheumatology centres. Little information exists on the clinical and serologic profiles of patients with sicca syndrome in tertiary rheumatology clinics, especially in ethnic groups such as Chinese, which includes both classified and unclassified forms of Sjögren's disease. The present study thus was designed to describe the clinical, laboratory and serological characteristics of patients with sicca syndrome and to explore the correlations of these characteristics with extraglandular manifestations. We hypothesized that there would be a relationship between the presence of certain autoimmune serologic abnormalities, specifically the presence of anti-SSA/Ro positivity and markers of systemic immune activation and a higher prevalence of extraglandular manifestations.

## 2 Methodology

### 2.1 Study design and setting

This was a retrospective observational cohort study performed in the Department of Rheumatology of a tertiary teaching hospital in China. The clinical records of consecutive adult inpatients who presented with sicca-related symptoms were checked from January 1, 2023, to December 31, 2025, using the hospital's electronic medical record (EMR) system. It is a major referral centre for autoimmune, rheumatic, and other diseases, and routinely assesses patients with chronic ocular and oral dryness for all rheumatological, immunologic, ophthalmological, and oral manifestations. The study aimed to define the clinical, laboratory, and serological profiles of patients with sicca syndrome and to assess the prevalence of autoimmune serological markers in this group. Data were collected from routinely maintained clinical records without affecting patient management or treatment decisions, and all data were extracted retrospectively.

Patients were specifically selected based on clinically documented sicca syndrome and not on the basis of a pre-existing diagnosis of primary Sjögren disease, so as to assess the wider population of patients who present for tertiary rheumatology practice with ocular and/or oral dryness. Classification for primary Sjögren disease was then determined by the 2016 American College of Rheumatology/European Alliance of Associations for Rheumatology (ACR/EULAR) classification criteria by using the variables available in the retrospective database, which included objective glandular variables and serologic variables. As a tertiary referral institution, the hospital receives patients referred from primary care, ophthalmology, oral medicine, and other medical specialties for persistent or diagnostically challenging sicca symptoms.

### 2.2 Study population and participant selection

The study population consisted of patients presenting to the rheumatology outpatient clinics for evaluation of persistent sicca symptoms and those admitted to the hospital during the study period for the same evaluation. All electronic medical records were carefully examined using diagnostic codes for sicca syndrome, xerophthalmia, xerostomia, autoimmune exocrinopathy, and associated rheumatologic diseases.

Initially, 1,024 patient records were found using the electronic database. Of these, 86 duplicate and repeated admissions were removed, and 938 unique patient records were subjected to detailed eligibility assessment. The 173 patients were then excluded for previously defined reasons: age below 18 years (*n* = 28), lack of clinical documentation to confirm sicca manifestations (*n* = 61), inadequate laboratory/serological investigations (*n* = 39), active malignancy or infectious disease (*n* = 21), previous head and neck radiotherapy or sicca-related side effects from medication (*n* = 15), or incomplete follow-up records, preventing baseline clinical data from being assessed (*n* = 9). After applying all the eligibility criteria, 765 patients formed the final study cohort and were included in the statistical analysis as shown in Figure 1.

**Figure 1:**
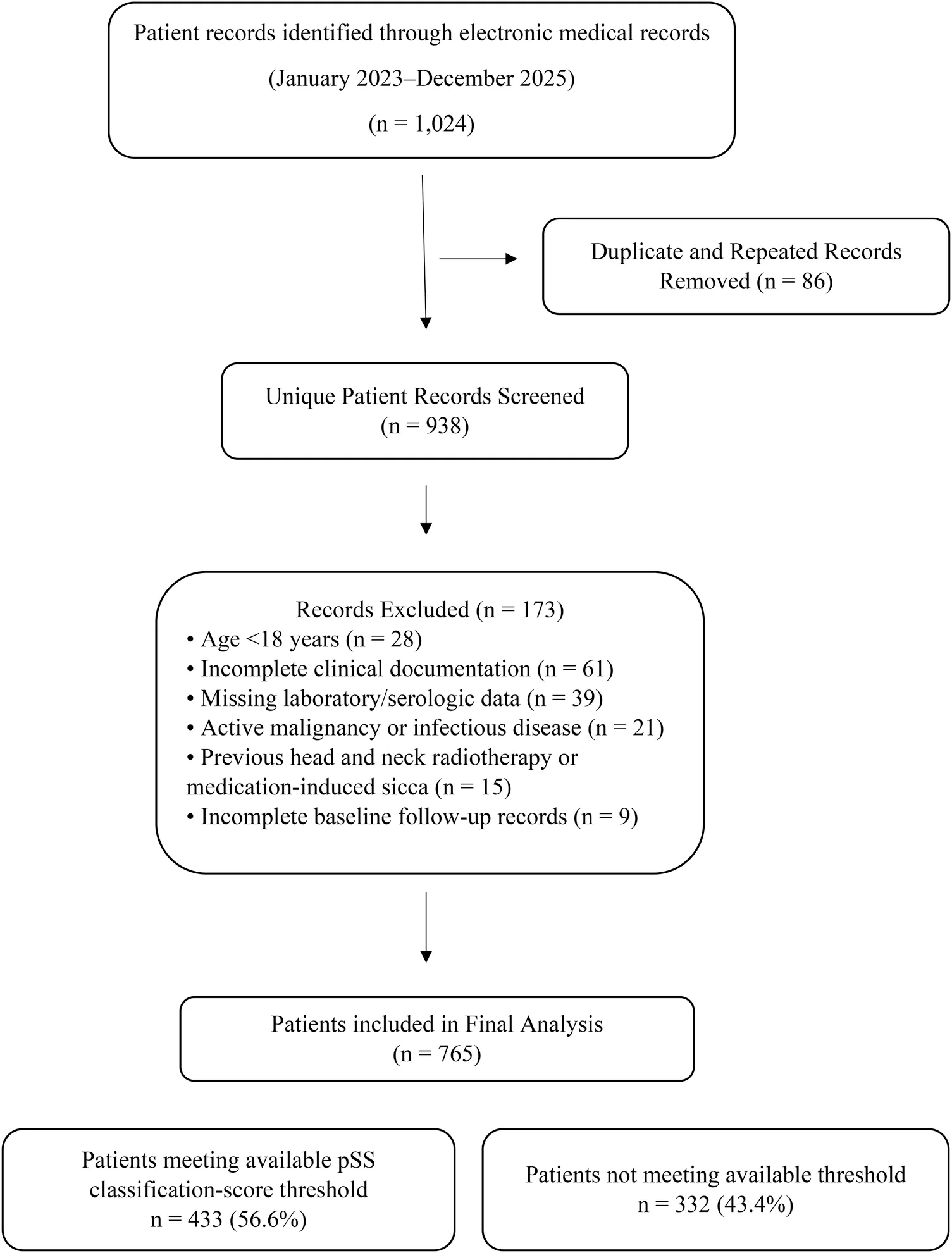
STROBE participant flow diagram.

### 2.3 Eligibility criteria

Patients were eligible if they were adults (aged 18 or older), had persistent ocular and/or oral dryness, and had undergone a comprehensive evaluation of their rheumatologic disease during the study period. Patients were eligible if they had all demographic data, detailed clinical assessment, laboratory tests performed as a routine, and a standardized autoimmune serologic testing available in the electronic medical records at the time of initial rheumatologic evaluation.

Patients whose medical records were incomplete, who had isolated transient symptoms of sicca associated with acute illness, prior radiation therapy of the head and neck, medication-induced xerostomia or xerophthalmia with no evidence of underlying rheumatologic disease, active malignancy, chronic infectious diseases that could have significant effects on immune status, pregnancy or missing critical laboratory or serologic variables for analyses were excluded. If a patient had more than one hospital visit in the study period, the first eligible visit was used for the patient, to eliminate the possibility of duplication.

### 2.4 Data collection

Two independent investigators used a standard electronic data abstraction form to extract clinical data retrospectively. Patient demographics were age at presentation, sex, BMI, smoking history, alcohol intake, duration of sicca symptoms, family history of autoimmune disease, and comorbid medical conditions.

The objective assessment of the glands was conducted as per the normal clinical routine and recorded in the EMRs. The Schirmer I test was used to assess lacrimal gland function in this group, using a filter-paper strip placed at the lower lateral lid of the eye without topical anaesthetic, and the wetting measured in millimetres after 5 min. For this study, a value of ≤5 mm/5 min was defined as abnormal. The ocular staining score was used to assess ocular surface damage and has been routinely reported by ophthalmology, as a score of corneal and conjunctival staining. The prespecified abnormal ocular staining threshold was applied for the clinical assessment and also for the calculation of the pSS classification score.

Whole salivary flow was measured without stimulation, by collecting saliva over a normal 15 min period, and measuring the volume collected in millilitres. Salivary flow reduction was classified as either present or absent, based on the institutional reference level for the duration of the study. Records of rheumatology/oral medicine were used to document additional oral findings, including oral candidiasis, dental caries, recurrent swelling of the parotid, and enlargement of the salivary glands. There was no routine use of minor salivary gland biopsy as part of the institutional evaluation pathway in the study period. As a result, there were no histopathological findings or focus scores available for the cohort and these were not imputed or inferred from other clinical or serologic variables. This was a retrospective study only, in which no further biopsy was done. When interpreting classification status this limitation was considered.

The following routine laboratory parameters were measured: complete blood count, erythrocyte sedimentation rate, C-reactive protein, liver and renal function tests, serum immunoglobulin concentrations, complement components, C3 and C4, serum albumin, and renal biochemical parameters. Routine clinical practice of autoimmune serologic testing involved testing for antinuclear antibodies, rheumatoid factor (RF), anti-SSA/Ro antibodies, anti-SSB/La antibodies, anticentromere antibodies, anti-double-stranded DNA antibodies, anti-U1-RNP antibodies, anti-Scl-70 antibodies, and other extractable nuclear antigen antibodies. Laboratory measurements were done during the study period from certified hospital laboratories using standardised automated immunologic assays.

Laboratory measurements were done during the study period from certified hospital laboratories using standardised automated immunologic assays. The routine use of minor salivary gland biopsy during the study period was not practiced, so there was no histopathological examination or focus-score data in the retrospective database. Since the study was conducted based on the clinical data already collected, no further biopsy was performed specifically for the study. As a result, focal lymphocytic sialadenitis and focus score were not available for all animals and were not used as analytical variables. Sicca syndrome was assessed accordingly based on the clinical manifestations, the objective examination of the ocular and oral glands and the available serologic results.

### 2.5 Operational definitions

The criteria for the diagnosis of sicca syndrome were: persistent subjective or objectively documented ocular dryness, oral dryness, or both, requiring a rheumatologic evaluation and documented in the medical record, regardless of the underlying autoimmune diagnosis.

Objective measurement of ocular and oral glandular dysfunction was based on the measurements obtained during routine examination. Any amount of wetting 5 mm or less after 5 min was considered an abnormal Schirmer I test. Ocular surface staining was defined as abnormal when the staining score recorded was equal to or exceeded the criteria of the clinical/classification system used. Unstimulated whole salivary flow was determined as decreased if the salivary volume measured in 15 min was below the institutional reference value for that period of the study. These measures were kept as objective measures of glandular dysfunction and evaluated separately from subjective reports of dryness in the eyes or mouth.

Dryness of the eye was categorised as chronic if dry eye symptoms persisted for at least 3 months or if objective evidence of tear break-up was demonstrated on ophthalmologic exam. Oral dryness was categorised as persistent xerostomia based on patient report or as a decrease in salivary secretion measured during clinical evaluation.

Primary Sjögren's syndrome (pSS) was assessed using the 2016 American College of Rheumatology/European Alliance of Associations for Rheumatology (ACR/EULAR) classification criteria for pSS, which were based on the clinical, objective glandular and serologic variables present in the retrospective database. Anti-SSA/Ro was associated with 3 points and a positive Schirmer test, with an ocular staining score of at least 10, and with the reduction of unstimulated salivary flow were associated with 1 point each. The available classification-score threshold was obtained by a cumulative score of ≥4. Minor salivary gland biopsy was not routinely used in the study period, and therefore the analysis did not equate the scores as a definitive diagnosis by the physician. Patients who had classification score available were thus described as “meeting the available pSS classification threshold” and those who did not reach the classification score were classified as “not meeting the available pSS classification threshold.” Both groups were still included in the overall group of patients with sicca syndrome.

Seropositivity for antinuclear antibody, anti-SSA/Ro antibody, anti-SSB/La antibody, rheumatoid factor, and anticentromere antibody was determined by the hospital's immunology department laboratory, based on the reference ranges in effect at the time of the antibody assays.

The presence of extra-glandular involvement was determined by documented involvement of organ systems other than the immune system, as determined by a rheumatologist's diagnosis of autoimmune disease: musculoskeletal, pulmonary, renal, neurological, dermatological, haematological, or vascular.

The inflammatory markers were considered elevated if their ESR or CRP levels exceeded the institutional upper reference limit. Hypocomplementemia was defined as serum C3 or C4 levels below the laboratory reference range. Laboratory reference values for immunoglobulin levels were used to define hypergammaglobulinemia.

### 2.6 Study outcomes

The main objective of the study was to define the demographic, clinical, laboratory and serologic profile of patients with sicca syndrome in a tertiary rheumatology centre.

Secondary end-points were the prevalence of major serologic markers of autoimmunity, the association between clinical features and serologic markers, the comparison of clinical features among patients with different antibody profiles, and the identification of independent factors associated with systemic extraglandular involvement, and assessment of the robustness of these associations using sensitivity analysis.

### 2.7 Statistical analysis

IBM SPSS Statistics version 29.0 (IBM Corp., Armonk, NY, USA) was used for all statistical analyses. The Shapiro–Wilk test was used to assess the normality of continuous variables. Values of normally distributed variables were reported as mean ± SD and analysed by the independent-samples Student's *t*-test or one-way analysis of variance, while those of non-normally distributed variables were reported as median (Q1, Q3) and analysed by the Mann–Whitney *U* test or Kruskal–Wallis test, as appropriate. Categorical variables were reported as frequencies and percentages and analysed by the Pearson chi-square test or Fisher's exact test when cell frequencies were small.

Pearson or Spearman correlation coefficients were used to assess the correlation between laboratory parameters and selected clinical variables, depending on the nature of the data. Predictors that were selected as candidates for multivariable logistic regression were chosen *a priori* for clinical relevance or from previous studies and also those variables with *P* < 0.10 from univariable analysis. Since several immunological variables were found to be biologically related, lack of multicollinearity was checked prior to developing the model with variance inflation factors (VIFs) and tolerance statistics. Those variables that were correlated were evaluated simultaneously and the final model was reduced to clinically relevant variables to avoid overadjustment and model parsimony. After controlling for demographic, clinical, inflammatory and immunological parameters, autoantibody variables remained as they did when they offered independent information. Odds ratios (ORs) with 95% confidence intervals (CIs) were presented. The c-statistic was used to measure model discrimination, and the Hosmer–Lemeshow goodness-of-fit test was used to measure model calibration. Sensitivity analyses involved re-estimating the model with various alternate model specifications without the highly correlated immunological predictors.

Multiple testing was taken into account, because of the multiple clinical, laboratory, serologic, subgroup and correlation analyses. The analyses were largely exploratory and hypothesis generating, and none of the analyses was formally adjusted for multiple comparisons, nor was any prespecified adjustment applied. For this reason, statistically significant results were evaluated based on the pattern of the results as a whole and viewed as associations, not causal or independent effects. A two-sided *P* value <0.05 was considered statistically significant.

### 2.8 Ethical considerations

This research was approved by the Ethics Committee of Guangzhou University of Chinese Medicine, Approval Number：K2025-176-02. The study was conducted in accordance with the ethical principles for research involving human participants, as stated in the Declaration of Helsinki and its amendments.

## 3 Results

### 3.1 Participant characteristics

The study included 765 patients with sicca syndrome. Among the 765 patients with sicca syndrome, 433 (56.6%) met the available 2016 ACR/EULAR classification-score threshold for primary Sjögren syndrome, whereas 332 (43.4%) did not meet the available threshold. Both groups were retained within the overall sicca syndrome cohort because the primary objective was to characterize the full clinical and serologic spectrum of sicca presentations rather than to restrict the analysis to classified primary Sjögren syndrome. The mean age of the cohort was 52.8 ± 13.6 years, with a high female-to-male ratio of 6.5:1. The majority of patients (61.7%) had a normal BMI, the median symptom duration was 24 months, and the median time to referral to rheumatology was 14 months. The most common presentation was combined ocular and oral dryness (70.7%), followed by isolated ocular dryness (15.8%) and isolated oral dryness (13.5%). The most common comorbidities were hypertension (26.9%), dyslipidaemia (19.5%), autoimmune thyroid disorder (15.4%) and osteoporosis (14.2%). Prior to referral, over half of patients used artificial tears (55.0%), and the majority were examined as outpatients (77.6%). Details of basic demographic and clinical data are shown in Table 1.

**Table 1:** Baseline demographic and clinical characteristics of patients with sicca syndrome (*N* = 765).

Variable	Total (*N* = 765)
Age (years), mean ± SD	52.8 ± 13.6
Age range (years)	18–84
Female sex, *n* (%)	663 (86.7)
Male sex, *n* (%)	102 (13.3)
Female-to-male ratio	6.5: 1
Height (cm), mean ± SD	162.1 ± 8.4
Weight (kg), mean ± SD	61.8 ± 11.7
BMI (kg/m^2^), mean ± SD	23.8 ± 3.9
BMI <18.5 kg/m^2^	39 (5.1)
BMI 18.5–24.9 kg/m^2^	472 (61.7)
BMI 25.0–29.9 kg/m^2^	201 (26.3)
BMI ≥30 kg/m^2^	53 (6.9)
Symptom duration (months), median (IQR)	24 (12–46)
Time to rheumatology referral (months), median (IQR)	14 (7–28)
Disease duration ≥5 years, *n* (%)	219 (28.6)
Rural residence, *n* (%)	284 (37.1)
Urban residence, *n* (%)	481 (62.9)
Current smoker, *n* (%)	71 (9.3)
Former smoker, *n* (%)	82 (10.7)
Current alcohol consumption, *n* (%)	95 (12.4)
Family history of autoimmune disease, *n* (%)	178 (23.3)
Previous ophthalmology consultation	212 (27.7)
Previous stomatology/oral medicine consultation	156 (20.4)
Referred from primary care	298 (39.0)
Referred from ophthalmology	177 (23.1)
Referred from oral medicine	94 (12.3)
Direct rheumatology consultation	196 (25.6)
Outpatient evaluation	594 (77.6)
Hospital admission	171 (22.4)
Ocular dryness only	121 (15.8)
Oral dryness only	103 (13.5)
Combined ocular and oral dryness	541 (70.7)
Hypertension	206 (26.9)
Type 2 diabetes mellitus	92 (12.0)
Autoimmune thyroid disease	118 (15.4)
Dyslipidemia	149 (19.5)
Osteoporosis	109 (14.2)
Chronic liver disease	37 (4.8)
Chronic kidney disease	28 (3.7)
Previous autoimmune diagnosis	167 (21.8)
Use of artificial tears before referral	421 (55.0)
Saliva substitutes before referral	168 (22.0)
Sicca syndrome classification according to available pSS criteria	
Met available 2016 ACR/EULAR classification-score threshold, *n* (%)	433 (56.6)
Did not meet available classification-score threshold, *n* (%)	332 (43.4)

Continuous variables are presented as mean ± standard deviation (SD) or median (interquartile range [IQR]), depending on the data distribution. Categorical variables are expressed as number (percentage). BMI, body mass index.

### 3.2 Sicca manifestations and extraglandular features

The clinical evaluation showed that eye dryness (86.5%) and oral dryness (84.2%) were the most common symptoms related to the glandular system; foreign-body sensation, use of artificial tears, and water intake during mealtimes were also very common (65.8%, 57.3%, and 61.6%, respectively). Objective glandular testing demonstrated substantial impairment of both lacrimal and salivary gland function. The mean Schirmer I test value was 4.8 ± 3.2 mm/5 min, and 514 patients (67.2%) had an abnormal Schirmer result according to the prespecified threshold. Ocular surface staining was abnormal in 463 patients (60.5%), with a median ocular staining score of 5 (IQR, 3–7). The median unstimulated salivary flow was 0.92 mL/15 min (IQR, 0.54–1.36), and reduced salivary flow was documented in 419 patients (54.8%). These findings are summarized in Table 2.

**Table 2:** Clinical manifestations and objective glandular assessment of patients with sicca syndrome (*N* = 765).

Variable	Total (*N* = 765)
Subjective glandular symptoms	
Ocular dryness	662 (86.5)
Oral dryness	644 (84.2)
Foreign-body sensation	503 (65.8)
Burning eyes	421 (55.0)
Blurred vision	286 (37.4)
Photosensitivity	192 (25.1)
Frequent use of artificial tears	438 (57.3)
Difficulty swallowing dry food	356 (46.5)
Frequent water intake during meals	471 (61.6)
Altered taste sensation	173 (22.6)
Hoarseness	96 (12.5)
Objective ocular assessment	
Schirmer *I* test (mm/5 min), mean ± SD	4.8 ± 3.2
Tear break-up time (seconds), mean ± SD	5.7 ± 2.8
Ocular staining score, median (IQR)	5 (3–7)
Abnormal Schirmer *I* test (≤5 mm/5 min), *n* (%)	514 (67.2)
Abnormal ocular surface staining	463 (60.5)
Objective oral assessment	
Unstimulated salivary flow (mL/15 min), median (IQR)	0.92 (0.54–1.36)
Reduced unstimulated salivary flow, *n* (%)	419 (54.8)
Dental caries	301 (39.3)
Oral candidiasis	69 (9.0)
Recurrent parotid swelling	132 (17.3)
Salivary gland enlargement	154 (20.1)
Constitutional manifestations	
Fatigue	358 (46.8)
Weight loss	38 (5.0)
Low-grade fever	52 (6.8)
Night sweats	21 (2.7)
Musculoskeletal manifestations	
Arthralgia	341 (44.6)
Inflammatory arthritis	216 (28.2)
Morning stiffness >30 min	187 (24.4)
Myalgia	176 (23.0)
Systemic manifestations	
Raynaud phenomenon	102 (13.3)
Cutaneous involvement	91 (11.9)
Vasculitic skin lesions	21 (2.7)
Peripheral neuropathy	63 (8.2)
Pulmonary involvement	57 (7.5)
Renal involvement	34 (4.4)
Thyroid involvement	109 (14.2)
Lymphadenopathy	46 (6.0)
Hepatic involvement	18 (2.4)
Hematologic involvement	42 (5.5)
ESSPRI score, mean ± SD	5.8 ± 1.7
Physician Global Assessment (0–10), mean ± SD	4.6 ± 1.8
Number of involved organ systems, median (IQR)	2 (1–3)
Patients with ≥1 extraglandular manifestation	452 (59.1)

### 3.3 Laboratory and serological characteristics

Systematic inflammation and immune dysregulation were identified in a significant number of patients through laboratory assessment. Haematological abnormalities were detected in 61.7% of patients having elevated ESR (>20 mm/h), 23.7% had elevated CRP (>10 mg/L), and 5.4% had decreased platelet count [<200,000/µL]. Immunological work-up showed increased IgG levels (43.9%) and, concurrently, decreased C3 (20.4%) and C4 (18.2%) complement levels. Moreover, 40.7% had vitamin D deficiency, and renal and hepatic biochemical parameters were essentially preserved. Characteristics of the study population, including laboratory and biochemical findings, are summarised in Table 3.

**Table 3:** Laboratory and biochemical characteristics of patients with sicca syndrome (*N* = 765).

Variable	Total (*N* = 765)
Hematological parameters	
Hemoglobin (g/L), mean ± SD	122.6 ± 15.8
Hemoglobin <120 g/L, *n* (%)	218 (28.5)
White blood cell count (×10⁹/L), mean ± SD	5.68 ± 1.71
Leukopenia (<4.0 × 10⁹/L), *n* (%)	132 (17.3)
Neutrophils (×10⁹/L), mean ± SD	3.46 ± 1.19
Lymphocytes (×10⁹/L), mean ± SD	1.61 ± 0.58
Platelet count (×10⁹/L), mean ± SD	246.8 ± 67.5
Thrombocytopenia, *n* (%)	41 (5.4)
Inflammatory markers	
ESR (mm/h), mean ± SD	34.7 ± 21.6
ESR >20 mm/h, *n* (%)	472 (61.7)
CRP (mg/L), median (IQR)	5.8 (2.9–10.6)
CRP >10 mg/L, *n* (%)	181 (23.7)
Biochemical parameters	
Serum albumin (g/L), mean ± SD	40.8 ± 4.7
Serum globulin (g/L), mean ± SD	35.2 ± 7.1
Albumin/Globulin ratio, mean ± SD	1.20 ± 0.29
Alanine aminotransferase (U/L), median (IQR)	23 (17–33)
Aspartate aminotransferase (U/L), median (IQR)	24 (18–32)
Serum creatinine (µmol/L), mean ± SD	71.3 ± 18.7
Estimated GFR (mL/min/1.73 m^2^), mean ± SD	91.6 ± 18.9
Blood urea nitrogen (mmol/L), mean ± SD	5.4 ± 1.6
Immunological parameters	
IgG (g/L), mean ± SD	18.6 ± 5.9
Elevated IgG, *n* (%)	336 (43.9)
IgA (g/L), mean ± SD	3.42 ± 1.24
IgM (g/L), mean ± SD	1.54 ± 0.82
Complement C3 (g/L), mean ± SD	0.93 ± 0.24
Low C3, *n* (%)	156 (20.4)
Complement C4 (g/L), mean ± SD	0.19 ± 0.08
Low C4, *n* (%)	139 (18.2)
Ferritin (ng/mL), median (IQR)	108 (63–187)
Vitamin D (ng/mL), mean ± SD	23.7 ± 8.6
Vitamin D deficiency, *n* (%)	311 (40.7)

Continuous variables are presented as mean ± standard deviation (SD) or median (interquartile range [IQR]), depending on the data distribution. Categorical variables are expressed as number (percentage). ESR, erythrocyte sedimentation rate; CRP, C-reactive protein; eGFR, estimated glomerular filtration rate; Ig, immunoglobulin.

### 3.4 Autoantibody and serologic profiles

A high prevalence of autoimmune markers was found in the cohort by serologic evaluation. The positivity of ANA was observed in 74.6% of the patients, high-titer ANA (≥1:320) in 41.4%, anti-SSA/Ro in 61.2%, anti-SSB/La in 36.1%, and rheumatoid factor in 44.7%. The presence of anticentromere (ACA) antibody was found in 10.6% of cases, while other extractable nuclear antibodies (ENAs) were relatively rare. There were also several patients with multiple autoantibody positivity (51.8% had at least two positive autoantibodies; 29.7% had three or more). In comparison, 14.1% of the patients were triple seronegative (ANA−/SSA−/SSB−). Details of the autoantibody and serologic findings of the study population are given in Table 4.

**Table 4:** Autoantibody and serologic characteristics of patients with sicca syndrome (*N* = 765).

Variable	Total (*N* = 765)
Antinuclear antibody (ANA)	
ANA positive	571 (74.6)
ANA titer 1:80	98 (12.8)
ANA titer 1:160	156 (20.4)
ANA titer ≥1:320	317 (41.4)
Anti-SSA/Ro antibody	
Positive	468 (61.2)
Negative	297 (38.8)
Anti-SSB/La antibody	
Positive	276 (36.1)
Negative	489 (63.9)
Rheumatoid factor (RF)	
RF positive	342 (44.7)
RF titer (IU/mL), median (IQR)	42 (18–96)
Anticentromere antibody (ACA)	
ACA positive	81 (10.6)
ACA negative	684 (89.4)
Other extractable nuclear antibodies	
Anti-RNP positive	83 (10.8)
Anti-Sm positive	39 (5.1)
Anti-Scl-70 positive	21 (2.7)
Anti-Jo-1 positive	9 (1.2)
Anti-dsDNA positive	28 (3.7)
Anti-centromere B positive	74 (9.7)
Autoantibody combinations	
ANA + SSA	382 (49.9)
ANA + SSA + SSB	214 (28.0)
ANA + RF	271 (35.4)
SSA + RF	236 (30.8)
SSA + SSB + RF	171 (22.4)
Triple seronegative (ANA−/SSA−/SSB−)	108 (14.1)
SSA−/SSB− phenotype	204 (26.7)
≥2 positive autoantibodies	396 (51.8)
≥3 positive autoantibodies	227 (29.7)

Values are presented as number (percentage) or median (interquartile range [IQR]). ANA, antinuclear antibody; RF, rheumatoid factor; ACA, anticentromere antibody; RNP, ribonucleoprotein; dsDNA, double-stranded DNA.

### 3.5 Comparison of clinical and laboratory characteristics according to anti-SSA/Ro antibody status

Patients with anti-SSA/Ro positivity were significantly younger, more often female and had a longer symptom duration compared to anti-SSA/Ro-negative patients. Patients with anti-SSA/Ro antibodies also had higher rates of salivary gland involvement, as evidenced by salivary flow, a positive Schirmer test, and higher rates of ocular and oral dryness. Moreover, the anti-SSA/Ro positive group had significantly higher rates of fatigue, arthralgia, inflammatory arthritis, Raynaud phenomenon, peripheral neuropathy, and pulmonary involvement, and at least one extraglandular manifestation. Anti-SSA/Ro positive patients had higher ESR, CRP and IgG levels, increased consumption of complement, positive ANA, anti-SSB and high levels of RF compared with negative anti-SSA/Ro patients (all *P* < 0.05). A detailed comparison of the demographic, clinical, laboratory and serologic features by anti-SSA/Ro antibody status is shown in Table 5.

**Table 5:** Comparison of demographic, clinical, laboratory, and serologic characteristics according to anti-SSA/Ro antibody status.

Variable	Anti-SSA/Ro positive (*n* = 468)	Anti-SSA/Ro negative (*n* = 297)	*P*-value
Age (years), mean ± SD	50.9 ± 12.8	55.7 ± 14.2	<0.001
Female sex, *n* (%)	417 (89.1)	246 (82.8)	0.013
Symptom duration (months), median (IQR)	28 (15–49)	19 (10–37)	<0.001
BMI (kg/m^2^), mean ± SD	23.4 ± 3.8	24.5 ± 4.0	0.001
Ocular dryness	416 (88.9)	246 (82.8)	0.017
Oral dryness	410 (87.6)	234 (78.8)	0.002
Positive Schirmer test	339 (72.4)	175 (58.9)	<0.001
Reduced salivary flow	285 (60.9)	134 (45.1)	<0.001
Fatigue	245 (52.4)	113 (38.0)	<0.001
Arthralgia	229 (48.9)	112 (37.7)	0.003
Inflammatory arthritis	150 (32.1)	66 (22.2)	0.004
Raynaud phenomenon	74 (15.8)	28 (9.4)	0.014
Peripheral neuropathy	48 (10.3)	15 (5.1)	0.015
Pulmonary involvement	42 (9.0)	15 (5.1)	0.048
ESR (mm/h), mean ± SD	38.9 ± 22.4	28.2 ± 18.7	<0.001
CRP (mg/L), median (IQR)	6.4 (3.2–11.4)	4.7 (2.4–8.2)	0.006
Hemoglobin (g/L), mean ± SD	120.8 ± 15.6	125.5 ± 15.8	<0.001
IgG (g/L), mean ± SD	20.3 ± 6.1	15.8 ± 4.8	<0.001
Low C3	121 (25.9)	35 (11.8)	<0.001
Low C4	111 (23.7)	28 (9.4)	<0.001
ANA positive	433 (92.5)	138 (46.5)	<0.001
Anti-SSB positive	245 (52.4)	31 (10.4)	<0.001
RF positive	248 (53.0)	94 (31.6)	<0.001
≥1 Extraglandular manifestation	305 (65.2)	147 (49.5)	<0.001

Continuous variables are presented as mean ± standard deviation (SD) or median (interquartile range [IQR]), whereas categorical variables are expressed as number (percentage). Comparisons were performed using the independent-samples *t*-test or Mann–Whitney *U* test for continuous variables and Pearson's chi-square test for categorical variables. Statistical significance was defined as *P* < 0.05.

### 3.6 Comparison according to anticentromere antibody (ACA) status

ACA-positive patients were significantly older and had a longer symptom duration as compared to ACA-negative patients. They also showed significantly higher rates of salivary gland enlargement, Raynaud phenomenon, involvement of skin and ANA positivity, while anti-SSA/Ro and anti-SSB/La antibodies were significantly lower (all *P* < 0.05). Additionally, patients with ACA were found to have lower ESR, CRP, and IgG levels than ACA-negative patients. The two groups were comparable on all parameters related to sex, BMI, glandular symptoms, objective glandular function, inflammatory arthritis, peripheral neuropathy, complement levels, rheumatoid factor (RF) positivity and overall prevalence of extraglandular manifestations. The clinical and laboratory features comparing the two groups, according to ACA status, are detailed in Table 6.

**Table 6:** Comparison of clinical and laboratory characteristics according to anticentromere antibody (ACA) status.

Variable	ACA Positive (*n* = 81)	ACA Negative (*n* = 684)	*P*-value
Age (years), mean ± SD	58.9 ± 11.2	52.1 ± 13.7	<0.001
Female sex	75 (92.6)	588 (86.0)	0.116
Symptom duration (months), median (IQR)	31 (18–56)	23 (12–44)	0.009
BMI (kg/m^2^), mean ± SD	24.1 ± 3.6	23.7 ± 3.9	0.432
Ocular dryness	72 (88.9)	590 (86.3)	0.526
Oral dryness	70 (86.4)	574 (83.9)	0.592
Positive Schirmer test	58 (71.6)	456 (66.7)	0.387
Reduced salivary flow	50 (61.7)	369 (53.9)	0.190
Salivary gland enlargement	25 (30.9)	129 (18.9)	0.012
Raynaud phenomenon	27 (33.3)	75 (11.0)	<0.001
Cutaneous involvement	19 (23.5)	72 (10.5)	0.001
Inflammatory arthritis	17 (21.0)	199 (29.1)	0.132
Peripheral neuropathy	8 (9.9)	55 (8.0)	0.571
Pulmonary involvement	10 (12.3)	47 (6.9)	0.083
ESR (mm/h), mean ± SD	29.6 ± 18.5	35.3 ± 21.8	0.031
CRP (mg/L), median (IQR)	4.5 (2.2–8.0)	6.0 (3.0–10.9)	0.042
Hemoglobin (g/L), mean ± SD	124.8 ± 15.2	122.3 ± 15.8	0.198
IgG (g/L), mean ± SD	16.5 ± 4.9	18.9 ± 5.9	<0.001
Low C3	10 (12.3)	146 (21.3)	0.062
Low C4	11 (13.6)	128 (18.7)	0.267
ANA positive	72 (88.9)	499 (73.0)	0.002
Anti-SSA/Ro positive	34 (42.0)	434 (63.5)	<0.001
Anti-SSB/La positive	18 (22.2)	258 (37.7)	0.006
RF positive	29 (35.8)	313 (45.8)	0.094
≥1 Extraglandular manifestation	43 (53.1)	409 (59.8)	0.268

Continuous variables are expressed as mean ± SD or median (IQR), while categorical variables are presented as number (percentage). Group comparisons were performed using independent-samples *t*-tests, Mann–Whitney *U* tests, Pearson's chi-square tests, or Fisher's exact tests, as appropriate. A two-sided *P* < 0.05 was considered statistically significant.

### 3.7 Factors associated with extraglandular manifestations

Multivariable logistic regression identified several factors that were independently associated with extraglandular manifestations. The strongest independent associations were observed for anti-SSA/Ro positivity, hypergammaglobulinemia, elevated ESR, and prolonged symptom duration. Other factors such as age, sex, BMI, positive Schirmer test, decreased salivary flow, antiANA and anti-centromere antibody positivity were not independently associated with extraglandular involvement. The multivariable logistic regression analysis results are shown in Table 7 and the adjusted odds ratios are shown as 95% confidence intervals in Figure 2.

**Table 7:** Primary Multivariable logistic regression analysis for factors associated with extraglandular manifestations in patients with sicca syndrome (*N* = 765).

Variable	Adjusted OR	95% CI	*P*-value
Age (per 10-year increase)	1.09	0.96–1.24	0.178
Female sex	1.28	0.82–2.01	0.281
BMI (per kg/m^2^ increase)	0.98	0.94–1.03	0.412
Symptom duration ≥24 months	1.73	1.23–2.44	0.002
Positive Schirmer test	1.18	0.84–1.66	0.336
Reduced salivary flow	1.31	0.95–1.81	0.098
Elevated ESR (>20 mm/h)	1.86	1.32–2.62	<0.001
Elevated CRP (>10 mg/L)	1.58	1.09–2.29	0.016
Hypergammaglobulinemia (Elevated IgG)	2.14	1.50–3.05	<0.001
Low Complement C3	1.81	1.22–2.67	0.003
Low Complement C4	1.69	1.13–2.52	0.011
ANA positivity	1.26	0.86–1.86	0.234
Anti-SSA/Ro positivity	2.47	1.73–3.52	<0.001
Anti-SSB/La positivity	1.62	1.12–2.35	0.010
Rheumatoid factor positivity	1.54	1.10–2.16	0.014
Anticentromere antibody positivity	0.88	0.53–1.47	0.621
Inflammatory arthritis	1.77	1.23–2.54	0.002
Fatigue	1.48	1.05–2.08	0.024

Variables were selected based on univariable associations (*P* < 0.10), clinical relevance, and previously reported associations. The primary model included demographic, clinical, inflammatory, and immunological variables. Multicollinearity was assessed using variance inflation factors; all VIFs were <2.5. Adjusted ORs are presented with 95% CIs.

**Figure 2:**
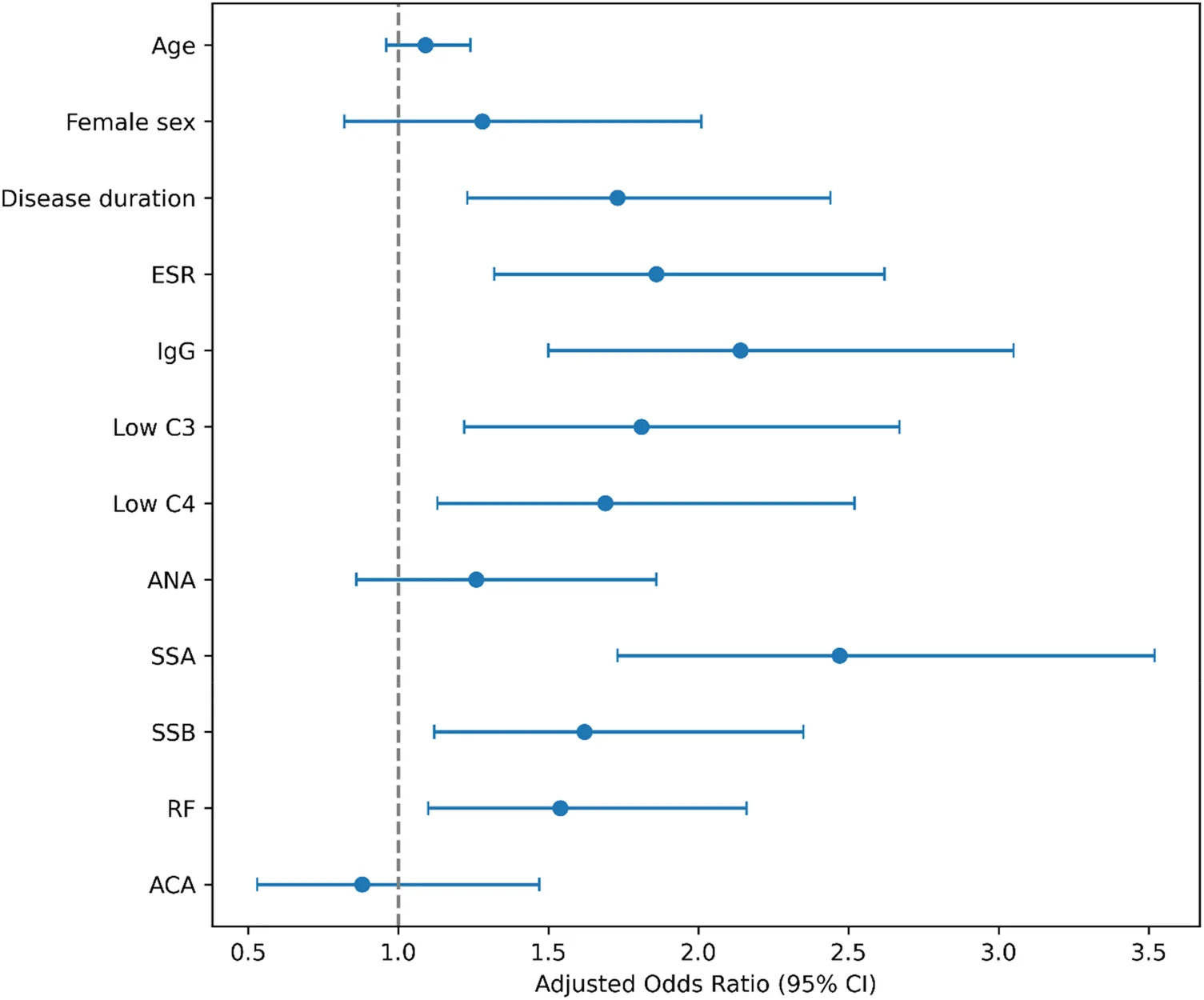
Forest plot of the primary multivariable logistic regression model for factors associated with extraglandular manifestations. The plot displays adjusted odds ratios (ORs) and corresponding 95% confidence intervals (CIs) for demographic, inflammatory, immunologic, and serologic variables included in the final multivariable logistic regression model. The vertical dashed line at OR = 1.0 represents the null value; estimates to the right indicate increased odds of extraglandular manifestations, whereas estimates to the left indicate decreased odds.

A multivariable sensitivity analysis with a reduced model was then conducted to examine the potential effects of correlated immunological variables and over adjustment effects. Clinically important demographic, glandular, inflammatory and serologic variables such as anti-SSA/Ro were included in the sensitivity model, while highly correlated serologic variables (ANA, anti-SSB/La, rheumatoid factor, IgG, C3, and C4) were not included. The reduced model was compared with the primary model with respect to the direction, magnitude, and statistical significance of the associations.

### 3.8 Sensitivity analysis of the multivariable regression model

Results of the sensitivity analysis were similar generally to the main multivariable model. Table 8 shows that, after the exclusion of highly correlated immunological factors, symptom duration of ≥24 months, elevated ESR, elevated CRP, anti-SSA/Ro positivity, inflammatory arthritis, and fatigue remained independently associated with extraglandular manifestations. In particular, there was a strong relationship between anti-SSA/Ro positivity and extraglandular involvement, with only minor changes in the adjusted effect estimate from the primary model. The direction and strength of the remaining clinically relevant associations were also broadly similar, indicating that the primary results were not due to the joint control of correlated serologic variables. Overall, the persistence of the principal associations after exclusion of potentially redundant immunological variables supports the robustness of the primary regression findings and suggests that the observed association between anti-SSA/Ro positivity and systemic involvement was not attributable solely to overadjustment by correlated serologic markers.

**Table 8:** Sensitivity analysis using a reduced multivariable logistic regression model for extraglandular manifestations.

Variable	Full model adjusted OR (95% CI)	Reduced model adjusted OR (95% CI)	*P* value*
Age, per 10-year increase	1.09 (0.96–1.24)	1.10 (0.97–1.25)	0.137
Female sex	1.28 (0.82–2.01)	1.25 (0.81–1.94)	0.311
BMI, per kg/m^2^ increase	0.98 (0.94–1.03)	0.99 (0.95–1.03)	0.458
Symptom duration ≥24 months	1.73 (1.23–2.44)	1.68 (1.21–2.34)	0.002
Positive Schirmer test	1.18 (0.84–1.66)	1.16 (0.84–1.62)	0.347
Reduced salivary flow	1.31 (0.95–1.81)	1.28 (0.94–1.75)	0.116
Elevated ESR	1.86 (1.32–2.62)	1.79 (1.29–2.49)	<0.001
Elevated CRP	1.58 (1.09–2.29)	1.53 (1.07–2.20)	0.021
Anti-SSA/Ro positivity	2.47 (1.73–3.52)	2.35 (1.68–3.29)	<0.001
Inflammatory arthritis	1.77 (1.23–2.54)	1.71 (1.21–2.42)	0.002
Fatigue	1.48 (1.05–2.08)	1.45 (1.04–2.03)	0.028
Anticentromere antibody positivity	0.88 (0.53–1.47)	0.91 (0.56–1.49)	0.683

* *P* values shown for the reduced model.

The reduced model demonstrated similar effect estimates to the primary model, with no material change in the direction or magnitude of the principal associations. Anti-SSA/Ro positivity remained independently associated with extraglandular manifestations. The reduced model showed acceptable discrimination (c-statistic approximately 0.79) and satisfactory calibration (Hosmer–Lemeshow *P* > 0.05). Similarity in the direction and magnitude of effect estimates between the primary and reduced models was considered evidence of model robustness.

### 3.9 Correlation between serologic markers and clinical variables

The correlation analyses demonstrated several positive associations between inflammatory markers, immunologic parameters, and selected clinical variables, although these findings should be interpreted cautiously because *P* values were not adjusted for multiple comparisons. Several clinical variables, including fatigue, arthralgia, inflammatory arthritis, impaired glandular function, ESSPRI score, and extraglandular manifestations, showed associations with ESR, CRP, IgG, low complement C3 and C4 levels, and anti-SSA/Ro antibodies. The strongest observed correlations involved ESR, anti-SSA/Ro, and IgG, whereas correlations involving anticentromere antibody were generally weak. Only weak correlations were observed with anticentromere antibody, with only a modest correlation with Raynaud phenomenon and with pulmonary involvement and limited correlations with other clinical parameters. The correlation coefficients are shown in Table 9 and the overall patterns of correlations are summarized in Figure 3.

**Table 9:** Correlation coefficients between serologic markers and selected clinical variables (*N* = 765).

Variables	ESR	CRP	IgG	Low C3	Low C4	Anti-SSA	RF	ACA
Fatigue	0.31***	0.22***	0.28***	0.19***	0.16***	0.27***	0.18***	0.05
Arthralgia	0.25***	0.19***	0.23***	0.15***	0.12**	0.24***	0.27***	0.03
Inflammatory arthritis	0.36***	0.28***	0.31***	0.22***	0.20***	0.29***	0.34***	−0.04
Positive Schirmer test	0.21***	0.14**	0.25***	0.18***	0.16***	0.38***	0.17***	0.08*
Reduced salivary flow	0.19***	0.12**	0.29***	0.17***	0.14**	0.35***	0.16***	0.06
Raynaud phenomenon	0.08*	0.05	0.09*	0.04	0.03	0.10**	0.06	0.34*
Pulmonary involvement	0.20***	0.17***	0.21***	0.15***	0.13**	0.18***	0.12**	0.16***
Peripheral neuropathy	0.17***	0.13**	0.18***	0.11**	0.09*	0.19***	0.15***	0.04
Extraglandular manifestations	0.41*	0.29*	0.37*	0.26*	0.23*	0.39*	0.28*	0.07
ESSPRI score	0.33***	0.24***	0.35***	0.22***	0.19***	0.31***	0.20***	0.05

ESR, erythrocyte sedimentation rate; CRP, C-reactive protein; IgG, immunoglobulin G; RF, rheumatoid factor; ACA, anticentromere antibody; ESSPRI, EULAR Sjögren's Syndrome Patient Reported Index. *P* values were not adjusted for multiple comparisons; therefore, nominally significant correlations should be interpreted cautiously and considered hypothesis-generating.

* *P* < 0.05.

** *P* < 0.01.

*** *P* < 0.001.

**Figure 3:**
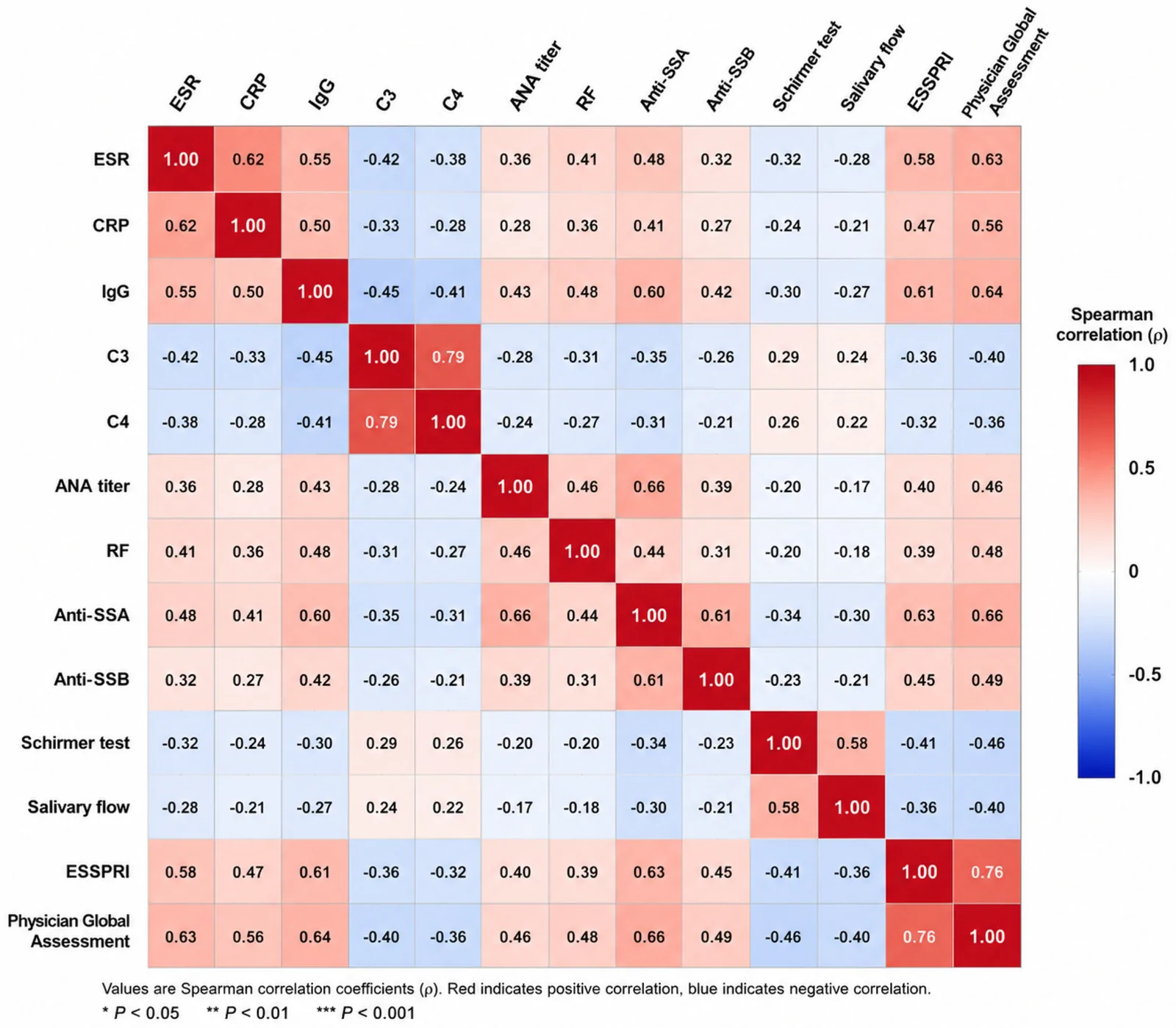
Correlation heatmap between serologic markers and clinical variables in patients with sicca syndrome (*N* = 765). Heatmap showing Spearman correlation coefficients (*ρ*) between inflammatory markers, immunologic biomarkers, autoantibodies, and selected clinical manifestations. Warmer colours indicate stronger positive correlations, whereas cooler colours indicate weaker or inverse associations. Numeric values within each cell represent the corresponding correlation coefficient.

### 3.10 Comparison of clinical, laboratory, and serologic characteristics according to primary Sjögren disease classification status

When comparing the analyses based on the available primary Sjögren disease classification according to the 2016 ACR/EULAR classification-score threshold, a clear clinical and immunologic profile emerged of patients who fulfilled the classification score. Compared to patients who were not classified as threshold, patients classified as threshold were younger, more likely to be female and had a longer duration of sicca symptoms. The objective glandular function was also worse, showing increased frequencies of abnormal Schirmer testing, ocular staining and reduced unstimulated salivary flow (all *P* < 0.001). Patients meeting the classification threshold had more frequent systemic manifestations, such as fatigue, arthralgia, inflammatory arthritis, peripheral neuropathy, and renal involvement (66.1% vs. 50.0%, *P* < 0.001). Higher ESR, CRP, IgG and hypocomplementemia frequency were the features of the immunologic profile. As predicted by the classification system, anti-SSA/Ro positivity was highly prevalent in the patients who crossed the threshold and the anti-SSB/La, ANA, and RF were also more likely to be positive. The detailed subgroup comparison is shown in Table 10.

**Table 10:** Comparison of demographic, clinical, laboratory, and serologic characteristics according to available primary Sjögren disease classification status.

Variable	Met available pSS classification threshold (*n* = 433)	Did not meet threshold (*n* = 332)	*P*-value
Demographic characteristics			
Age, years, mean ± SD	50.8 ± 12.7	55.4 ± 14.1	<0.001
Female sex, *n* (%)	387 (89.4)	276 (83.1)	0.018
BMI, kg/m^2^, mean ± SD	23.5 ± 3.7	24.2 ± 4.1	0.014
Symptom duration, months, median (IQR)	27 (14–48)	20 (10–40)	<0.001
Symptom duration ≥24 months, *n* (%)	238 (55.0)	142 (42.8)	0.001
Glandular manifestations			
Ocular dryness, *n* (%)	388 (89.6)	274 (82.5)	0.006
Oral dryness, *n* (%)	381 (88.0)	263 (79.2)	0.001
Foreign-body sensation, *n* (%)	302 (69.7)	201 (60.5)	0.011
Frequent artificial tear use, *n* (%)	266 (61.4)	172 (51.8)	0.009
Difficulty swallowing dry food, *n* (%)	225 (52.0)	131 (39.5)	0.001
Recurrent parotid swelling, *n* (%)	91 (21.0)	41 (12.3)	0.004
Salivary gland enlargement, *n* (%)	103 (23.8)	51 (15.4)	0.006
Objective glandular assessment			
Schirmer I test, mm/5 min, mean ± SD	4.3 ± 2.9	5.4 ± 3.5	<0.001
Positive Schirmer test, *n* (%)	326 (75.3)	188 (56.6)	<0.001
Ocular staining score, median (IQR)	6 (4–8)	4 (2–6)	<0.001
Positive ocular staining, *n* (%)	292 (67.4)	171 (51.5)	<0.001
Unstimulated salivary flow, mL/15 min, median (IQR)	0.74 (0.43–1.10)	1.12 (0.68–1.55)	<0.001
Reduced salivary flow, *n* (%)	275 (63.5)	144 (43.4)	<0.001
Constitutional and musculoskeletal manifestations			
Fatigue, *n* (%)	226 (52.2)	132 (39.8)	0.001
Arthralgia, *n* (%)	212 (48.9)	129 (38.9)	0.006
Inflammatory arthritis, *n* (%)	137 (31.6)	79 (23.8)	0.022
Morning stiffness >30 min, *n* (%)	119 (27.5)	68 (20.5)	0.030
Myalgia, *n* (%)	107 (24.7)	69 (20.8)	0.217
Systemic manifestations			
Raynaud phenomenon, *n* (%)	68 (15.7)	34 (10.2)	0.032
Cutaneous involvement, *n* (%)	61 (14.1)	30 (9.0)	0.039
Peripheral neuropathy, *n* (%)	44 (10.2)	19 (5.7)	0.026
Pulmonary involvement, *n* (%)	39 (9.0)	18 (5.4)	0.075
Renal involvement, *n* (%)	27 (6.2)	7 (2.1)	0.006
Lymphadenopathy, *n* (%)	32 (7.4)	14 (4.2)	0.068
Thyroid involvement, *n* (%)	73 (16.9)	36 (10.8)	0.023
≥1 extraglandular manifestation, *n* (%)	286 (66.1)	166 (50.0)	<0.001
Number of involved organ systems, median (IQR)	2 (1–3)	1 (0–2)	<0.001
Inflammatory and immunologic laboratory findings			
ESR, mm/h, mean ± SD	38.1 ± 22.0	30.2 ± 19.8	<0.001
ESR >20 mm/h, *n* (%)	294 (67.9)	178 (53.6)	<0.001
CRP, mg/L, median (IQR)	6.2 (3.1–11.3)	5.2 (2.7–9.4)	0.018
CRP >10 mg/L, *n* (%)	117 (27.0)	64 (19.3)	0.018
Hemoglobin, g/L, mean ± SD	120.9 ± 15.4	124.8 ± 15.9	0.001
IgG, g/L, mean ± SD	20.1 ± 6.0	16.7 ± 5.0	<0.001
Elevated IgG, *n* (%)	223 (51.5)	113 (34.0)	<0.001
Low C3, *n* (%)	106 (24.5)	50 (15.1)	0.002
Low C4, *n* (%)	98 (22.6)	41 (12.3)	<0.001
Autoantibody profile			
ANA positive, *n* (%)	412 (95.2)	159 (47.9)	<0.001
ANA ≥1:320, *n* (%)	238 (55.0)	79 (23.8)	<0.001
Anti-SSA/Ro positive, *n* (%)	407 (94.0)	61 (18.4)	<0.001
Anti-SSB/La positive, *n* (%)	227 (52.4)	49 (14.8)	<0.001
RF positive, *n* (%)	241 (55.7)	101 (30.4)	<0.001
Anticentromere antibody positive, *n* (%)	40 (9.2)	41 (12.3)	0.177
Anti-RNP positive, *n* (%)	51 (11.8)	32 (9.6)	0.355
Anti-dsDNA positive, *n* (%)	17 (3.9)	11 (3.3)	0.654
≥2 positive autoantibodies, *n* (%)	283 (65.4)	113 (34.0)	<0.001
≥3 positive autoantibodies, *n* (%)	175 (40.4)	52 (15.7)	<0.001
ESSPRI score, mean ± SD	6.1 ± 1.6	5.4 ± 1.8	<0.001

pSS, primary Sjögren disease; BMI, body mass index; IQR, interquartile range; SD, standard deviation; ESR, erythrocyte sedimentation rate; CRP, C-reactive protein; IgG, immunoglobulin G; C3/C4, complement components 3 and 4; ANA, antinuclear antibody; RF, rheumatoid factor; RNP, ribonucleoprotein; dsDNA, double-stranded DNA; ESSPRI, EULAR Sjögren's Syndrome Patient Reported Index. Continuous variables are presented as mean ± SD or median (IQR), according to distribution. Categorical variables are presented as *n* (%). Comparisons were performed using independent-samples *t*-test or Mann–Whitney *U* test for continuous variables and Pearson *χ*^2^ test or Fisher's exact test for categorical variables, as appropriate. Statistical significance was defined as two-sided *P* < 0.05.

## 4 Discussion

The current tertiary Chinese population with sicca syndrome showed a high percentage of female patients, a middle age at onset of the disease, a long duration of symptoms leading to presentation to the rheumatology clinic and a high presence of associated comorbidities. In our patients, there was a ratio of 6.5:1 for females to males, which is slightly lower than the male to female ratio of approximately 9:1–14:1 observed in other established Sjögren patient cohorts. This difference was noted in the broader inclusion criteria of the present study, which included patients with clinically documented sicca syndrome, and not only patients with a diagnosis or clinical classification of primary Sjögren disease. Indeed, 332 patients (43.4%) did not meet the available 2016 ACR/EULAR classification-score threshold for primary Sjögren disease, indicating that a substantial proportion of the cohort represented sicca presentations beyond the classified primary Sjögren disease population. Inclusion of these non-classified sicca patients may partly explain the less pronounced female predominance compared with cohorts restricted to primary Sjögren disease.

Patients included in our study were those with sicca syndrome, not only those diagnosed as having primary Sjögren disease; thus, the results should be interpreted with caution when comparing with established cohorts of primary Sjögren disease. A large clinical and serologic heterogeneity was seen in the total sicca population, although certain clinical and serologic characteristics that were observed among those who were classified as meeting the available classification threshold had been reported in primary Sjögren disease cohorts. The picture of female predominance and multiple comorbidities was also noted by Tarn et al. (14) and was indicative of the clinically challenging circumstances that can be seen in the majority of patients seen in a typical rheumatology setting when evaluating for autoimmune exocrine disease.

Similarly, Zhang et al. (15) found that disease duration correlated with clinical phenotype and systemic involvement in primary Sjögren syndrome, and in our cohort, disease onset was followed by a longer symptom duration before specialist evaluation. Overall, several features observed in our tertiary-care sicca cohort overlap with those reported in autoimmune sicca and primary Sjögren disease populations; however, these similarities should not be interpreted as evidence that the two populations are equivalent. It is important to also interpret these findings in the context of the tertiary-care setting. Patients referred to a specialist rheumatology centre are more likely to have persistently severe sicca symptoms or symptoms that are difficult to diagnose or suspected to be autoimmune than those managed in community or primary care. This referral pattern may therefore, partly, account for the relatively high prevalence of systemic features and serologic abnormalities seen in our patients, and for the difficulty of directly generalizing our observations to the larger population of patients with sicca symptoms.

Importantly, the cohort was selected mainly based on clinically documented sicca symptoms and not based on a diagnosis of primary Sjögren disease (pSS). The percentage that also met the 2016 ACR/EULAR classification criteria for pSS must be viewed as a subset of the sicca population corresponding to those that were autoimmune. This is significant as anti-SSA/Ro antibodies, hypergammaglobulinemia, hypocomplementemia and systemic manifestations are frequent in this group and may represent a significant autoimmune phenotype. However, because some patients were not classified as pSS, the study included a range of clinical and serological heterogeneity that is seen in tertiary-care patients who present with sicca symptoms. Referral characteristics may also have contributed to the observed sex distribution, as the study was conducted in a tertiary rheumatology centre receiving patients from primary care, ophthalmology, oral medicine, and other specialties. Differences in referral pathways and healthcare-seeking patterns may influence the demographic composition of a tertiary-care sicca cohort. Finally, population-specific demographic and healthcare characteristics may contribute to differences in sex distribution between Chinese tertiary-care sicca populations and previously reported international Sjögren disease cohorts; however, the present study was not designed to determine the relative contribution of these factors.

The combination of eye and mouth dryness, together with the high prevalence of fatigue, musculoskeletal symptoms, and extraglandular manifestations among our sicca patients, is consistent with the very diverse clinical presentation usually seen in tertiary rheumatology practice. We did not limit the study to primary Sjögren patients, but our findings in terms of glandular and systemic involvement are very similar to what has been reported in autoimmune sicca patients. Basheva-Kraeva et al. (16) noted that sicca patients can present with a variety of clinical manifestations, both seropositive and seronegative, making the diagnosis of sicca syndrome very complex. Likewise, fatigue and arthralgia, and the increasing extraglandular involvement, have been reported as common symptoms in the clinical spectrum of Sjögren disease, and neurological, pulmonary, renal and cutaneous manifestations are considered to significantly contribute to disease heterogeneity, respectively, by Manfrè et al. (17) and Mihai et al. (18). The similarities imply that, irrespective of the final diagnosis, the patient with sicca syndrome should be comprehensively evaluated systemically.

The laboratory profile of our cohort, including raised inflammatory markers, hypergammaglobulinemia, decreased complement levels and haematological abnormalities, is in general very similar to that of autoimmune sicca disorders. These cell markers have been primarily studied in primary Sjögren syndrome, but analogous immune abnormalities can also be seen in the clinically symptomatic sicca patients. While Berardicurti et al. (19) showed that serologic abnormalities correlate with salivary gland inflammatory changes, André ET AL (20), showed that inflammatory indices, complement consumption, and immunoglobulin abnormalities are important indicators of systemic immune activation. In addition, the high level of IgG in our cohort of patients is similar to findings from other studies showing that sustained B-cell activation is a key mechanism underlying chronic autoimmunity and autoantibody production in Sjögren disease, as summarised by Du et al. (21) These findings together warrant the importance of evaluating the immune system in the workup of patients with sicca syndrome.

The prevalence of ANA and anti-SSA positivity combined with several combinations of autoantibodies found in our cohort also support the association that significant autoimmune component is present in the group of patients who present with sicca symptoms. These serologic markers have previously been described in classified primary Sjögren syndrome, but our results show that the immunologic profiles of tertiary sicca populations are similar. Despite the emergence of novel biomarkers, conventional autoantibodies still play a role in disease characterisation, as noted by Cafaro et al. (22). Building on this, Engelke et al. (23) reported that emerging autoantibodies could enhance diagnostic stratification over traditional serologic testing. Baer et al. (24), however, found that anti-SSB antibody alone is not closely linked to the classical Sjögren phenotype, and Tsaliki et al. (25) suggested that patients with sicca symptoms but no conventional autoantibodies are likely to represent distinct immunological phenotypes that can be detected using novel salivary autoantibodies.

Anti-SSA-positive patients in our sicca cohort were younger, had a longer disease duration, had more severe glandular dysfunction, were more inflammatory and had a higher prevalence of extraglandular findings than anti-SSA-negative patients, suggesting that anti-SSA positivity is associated with a more active autoimmune phenotype in patients with sicca symptoms. While primarily described in primary Sjögren's, our findings are similar to those reported in the literature. Vitali et al. (26) highlighted that anti-SSA positivity correlates with disease activity and a wider systemic involvement, and Chatzis et al. (27) suggested serologic stratification as a cornerstone of precision medicine strategies in the care of autoimmune exocrinopathies. Similarly, Luo et al. (28) found that early disease onset patients were more immunologically activated and had more extensive clinical symptoms, and Marketos et al. (29), noted that seronegative patients with sicca symptoms could represent an important group to consider, even if they lack conventional Sjögren-specific autoantibodies. These findings support the clinical relevance of anti-SSA/Ro positivity as a marker associated with a more pronounced autoimmune and systemic clinical phenotype in patients presenting with sicca symptoms.

A higher age, more frequent Raynaud phenomenon, and salivary gland enlargement were present in the anticentromere antibody-positive group compared with the anti-SSA-, anti-SSB-, and IgG-positive groups, suggesting a distinct serologic and clinical pattern within the sicca spectrum. This is similar to the results of other studies of autoimmune sicca disorders, which have found that patients who are positive for ACA have typical vascular findings and immunologic characteristics. In a study of 100 patients with ACA antibodies, Park et al. (30) and associates noted a distinctive clinical presentation: Raynaud phenomenon was present, and the other serologic patterns were not typical of Sjögren's. In another study of 32 patients with ACA, which were distinct from the typical Sjögren serologic pattern, Li et al. (31), also found clinically relevant sicca symptoms. Recently, Liu et al. (32) showed that patients with ACA exhibit distinct clinical and immunological traits, further suggesting that ACA represents a distinct subset of autoimmune patients with sicca symptoms.

In our multivariable analysis, long duration of symptoms, elevated inflammatory markers, hypergammaglobulinemia, hypocomplementemia, anti-SSA and anti-SSB positivity, rheumatoid factor positivity, inflammatory arthritis and fatigue were independently associated with extraglandular manifestations, reflecting the strong association between systemic immune activation and disease expression in sicca syndrome. The results of this study are similar to those of Zhang et al. (33), who found serologic abnormalities as important correlates of systemic manifestations in primary Sjögren syndrome. Likewise, Koh et al. (34) reported that hypergammaglobulinemia correlated with a higher disease burden of the glandular and extraglandular features, and Jordán-González et al. (35) showed that hypocomplementemia was associated with a more active immunologic phenotype and higher systemic involvement. Taken together, these findings support the association of inflammatory and immunologic abnormalities with systemic extraglandular manifestations in patients with sicca syndrome.

Importantly, sensitivity analysis with a reduced regression model gave broadly similar estimates even though potentially correlated immunological variables were removed. This finding was also strong because anti-SSA/Ro positivity persisted with extraglandular manifestations, making it unlikely that such association was only due to overadjustment. However, due to the observational design, no causal interpretation is possible, and residual confounding by unmeasured clinical or immunological factors cannot be ruled out. Similarly, the more frequent rheumatoid factor positivity observed among anti-SSA/Ro-positive patients (53.0% vs. 31.6%, *P* < 0.001) was assessed cross-sectionally and should be regarded as a co-occurrence (association), possibly reflecting shared B-cell hyperactivity, rather than as evidence that one autoantibody causes the other or causes extra glandular involvement.

Several correlations between inflammatory markers, immunologic abnormalities, autoantibody profiles, glandular dysfunction, symptom severity and extraglandular manifestations were observed. The results indicate that immune dysfunction increases with increasing clinical burden in patients with sicca syndrome, although no multiplicity adjustment was made for the multiple comparisons, each correlation should be regarded as exploratory and interpreted with caution. These results are similar to those of the integrated biomarker approach described by Lodba et al. (36), who showed that the combination of clinical and serologic profile is helpful in the characterisation of autoimmune sicca disease. Similarly, Lackner et al. (37) noted a relationship between symptom burden and severity, with a strong correlation between dryness and objective measurements of lacrimal and salivary gland dysfunction. Moreover, Li et al. (38) reported associations between inflammatory markers and clinical disease characteristics, supporting the potential relevance of serologic measurements for clinical assessment in patients with sicca syndrome. However, the present cross-sectional analysis does not establish whether these markers are associated with subsequent disease progression or long-term outcomes.

### 4.1 Strengths of the study

There are several strengths in this study. First, it included a relatively large number of patients with sicca syndrome (765) who were evaluated at a tertiary rheumatology centre, providing a comprehensive demographic, clinical, laboratory, and serological profile of this heterogeneous group. Second, the study combined subjective symptoms with objective evaluations of the glands, extensive immunological evaluations and multivariable statistical analysis, allowing a comprehensive assessment of factors associated with systemic manifestations. Lastly, the definition of antibody-defined subgroups and the correlation between antibody markers and clinical features provide clinically relevant information, which could be useful for clinical characterization and the evaluation of patients in routine rheumatology practice with sicca syndrome.

### 4.2 Limitations of the study

The study design is retrospective, single centre which leads to a risk of selection and referral bias. Patients were referred to the institution, and therefore, a higher pre-test probability of autoimmune disease, more systemic manifestations, more persistent sicca symptoms or more diagnostic uncertainty could have existed compared to patients treated in general outpatient care or primary care. This referral pattern could account for some of the high serologic abnormalities that were autoimmune in this population and may preclude direct extrapolation of the results to other community-based or non-rheumatology sicca cohorts. Further, those with milder or self-limited sicca symptoms may have been under-represented as they may not have reached a tertiary rheumatology service. Therefore, the prevalence rates observed should be considered representative of a tertiary care rheumatologic sicca population, not the general Chinese population with sicca symptoms. Because the study was retrospective, the observed associations should not be interpreted as evidence of temporal relationships, causality, prognostic value, or prediction of future extraglandular manifestations or other clinical outcomes. Because the cohort was defined as sicca syndrome rather than exclusively as pSS, clinical and serologic heterogeneity may have influenced the observed associations, and classification criteria should not be interpreted as diagnostic criteria for individual patients.

Systematic minor salivary gland biopsy data was another limitation for diagnosis. Histopathological evidence of focal lymphocytic sialadenitis and focus scores were unable to be included in the analysis due to the fact that biopsies were not routinely performed during the study period. This is especially true when evaluating pSS classification status as some patients with sicca symptoms may have objective or serologic markers suggestive of an autoimmune disease, but lack histopathologic evidence. However, it used several of the standard, commonly available physical tests for glandular dysfunction as well—Schirmer test, staining of the ocular surface and unstimulated salivary flow—in addition to a comprehensive serologic evaluation. Therefore, the distinction between classified primary Sjögren disease and non-classified sicca syndrome should be interpreted as a classification based on the available clinical, objective glandular, and serologic information rather than as a definitive etiologic distinction for every participant.

In addition, several serologic and immunological variables are biologically interrelated, creating a potential risk of multicollinearity and overadjustment in multivariable models. Although VIF assessment and sensitivity analysis using a reduced model demonstrated broadly stable estimates, residual model dependence cannot be completely excluded. Also, there are numerous clinical, laboratory, serologic, subgroup, and correlation comparisons, which also may lead to multiple testing type I error. There was no formal multiplicity adjustment in any of the analyses because they were mainly exploratory. Therefore, results of any nominally significant findings must be taken with some circumspection and within the context of the overall consistency of the associations observed.

### 4.3 Future recommendations

Further prospective multicenter studies with larger and more diverse patient populations are warranted to confirm these findings and improve their generalizability. Longitudinal studies are also needed to determine whether the clinical and serologic associations identified in the present study are related to subsequent changes in disease manifestations, extraglandular involvement, and long-term clinical outcomes. Future studies incorporating more detailed immunologic, molecular, imaging, and genomic markers may further refine sicca syndrome characterization and improve understanding of disease heterogeneity. Such studies may also help determine whether specific serologic profiles have clinically meaningful implications for long-term monitoring and management.

## 5 Conclusion

Overall, this study provides a descriptive characterization of the clinical and serological features of patients with sicca syndrome in a single tertiary-care cohort in China. Anti-SSA/Ro positivity was associated with a distinct serological and potentially systemic phenotype, characterized by more frequent hypergammaglobulinemia, hypocomplementemia, rheumatoid factor positivity and extra glandular manifestations. Because of the retrospective, cross-sectional design, these relationships, including the co-occurrence of anti-SSA/Ro and rheumatoid factor positivity, should be interpreted as associations rather than causal effects; the data do not indicate whether one autoantibody influences the other or whether either leads to extra glandular involvement. Clinically, anti-SSA/Ro positivity may help identify patients who warrant closer assessment for systemic involvement. Combining careful clinical evaluation with comprehensive serologic testing may therefore help inform, rather than determine, the individualized assessment and follow-up of patients with sicca syndrome. Prospective longitudinal studies are needed to confirm these associations and to establish their prognostic and therapeutic relevance.

## Data Availability

The raw data supporting the conclusions of this article will be made available by the authors, without undue reservation.
